# Isolation and Identification of Severe Fever with Thrombocytopenia Syndrome Virus from Farmed Mink in Shandong, China

**DOI:** 10.1155/2024/9604673

**Published:** 2024-04-05

**Authors:** Xiangshu Meng, Jian Sun, Mengfan Yao, Yue Sun, Han Xu, Chao Liu, Han Chen, Jie Guo, Xiaoxuan Nie, Longbin He, Zongzheng Zhao, Nan Li, Zekun Wang, Jianke Wang

**Affiliations:** ^1^Hebei Veterinary Biotechnology Innovation Center, College of Veterinary Medicine, Hebei Agricultural University, Baoding, China; ^2^Weihai Ocean Vocational College, Rongcheng, China; ^3^Changchun Veterinary Research Institute, Chinese Academy of Agricultural Sciences, Changchun, China; ^4^Joint National Laboratory for Antibody Drug Engineering, The First Affliated Hospital, Henan University, Kaifeng, Henan, China

## Abstract

Severe fever with thrombocytopenia syndrome (SFTS) virus, recently named *Bandavirus dabieense*, belongs to the genus *Bandavirus* of family *Phenuiviridae*, and it causes SFTS in humans with clinical symptoms including fever, thrombocytopenia, gastrointestinal symptoms, and leukocytopenia. However, there are few reports on the pathogenesis of SFTSV in animals. This study first isolated the SFTSV strain SD22-2 from sick-farmed mink. Viral metagenomics was used to detect SFTSV nucleotide in the clinical specimens obtained from symptomatic minks. Then, we isolated the virus using Vero and DH82 cells, and Real-Time Quantitative PCR (RT-qPCR), indirect immunofluorescence assay, transmission electron microscopy, and Western blotting identified it. Meanwhile, phylogenetic analysis based on partial L, M, and S segment sequences indicated that the mink-origin SFTSV strain SD22-2 belonged to genotype D and was genetically close to the HB2016-003 strain isolated from humans. Taken together, we isolated and identified an SFTSV from farmed mink that may be the reservoir hosts of SFTSV. We should pay more attention to farmed minks and biosecurity practices, and active surveillance at fur farms must be reviewed and enhanced.

## 1. Introduction

Severe fever with thrombocytopenia syndrome (SFTS), caused by a bunyavirus [SFTS virus (SFTSV)] that was recently named *Bandavirus dabieense* in the family *Phenuiviridae*, is an emerging tick-borne viral zoonosis which posed a health threat to humans and animals [1]. SFTSV is a novel important pathogen, causing clinical symptoms such as fever, thrombocytopenia, gastrointestinal symptoms, and leukocytopenia in humans, with a high fatality rate of up to 30% [2, 3]. SFTS was first identified in China in 2009. Now it has been reported in at least nine countries, including China [2], Japan [4], South Korea [5], Vietnam [6], Myanmar [7], Pakistan [8], United Arab Emirates [9], Thailand [10], and United States [11], meaning it has rapidly expanded geographic distribution.

Recently, many studies have indicated that SFTSV infected domesticated and wild animals in some Asian countries [12–15]. Only viral RNA and anti-SFTSV antibodies have been observed among domesticated and wild animals, including sheep, cattle, dogs, cats, pigs, chickens, deer, wild boars, minks, yellow weasels, hares, elk, shrews, and raccoons [12–18]. Still, no SFTSVs were isolated from animals except sheep, cattle, and dogs [14]. Unfortunately, there is no report about the pathogenesis of SFTSV on farmed minks.

As with other viruses in the family *Phenuiviridae*, SFTSV is an enveloped negative-sense single-stranded RNA virus whose genome contains three segments. The segments of large (L), medium (M), and small (S) encode an RNA-dependent RNA polymerase, surface glycoproteins (Gn and Gc), nucleoprotein (NP), and nonstructural (NSs) proteins, respectively [3]. SFTSV has been classified into six genotypes (A, B, C, D, E, and F) based on segments L, M, and S sequences, and all of the six genotypes are prevalent in China [16, 19].

Fur animals are commonly bred in the East and North of China, especially in Shandong Province. In the present study, high-throughput sequencing technologies were applied to specifically explore the viral communities in sick minks and we isolated an SFTSV, named SD22-2, from sick farmed mink in Shandong, China. To our knowledge, this is the first pathogenesis of SFTSV study on minks.

## 2. Materials and Methods

### 2.1. Cases Report and Sample Collection

Every June to November of years from 2020 to 2023, many minks died from an outbreak of disease with unclear cause in Haiyang City, which is a county-level city in Yantai city, Shandong province in eastern China (120°50′–121°29′E, 36°16′–37°10′N; *Supplementary 1*). This disease affects all ages of mink, with an incidence rate of 30%–41% and a mortality rate of 100%. When minks lost appetite, they were found dead after 1–3 days. Typical clinical signs of minks are fever, diarrhea with black or yellow soft feces, enlarged and congested lymph nodes, enlarged spleen with infarction and severe hemorrhage, and gastrointestinal bleeding (*Supplementary 1*). Haiyang is one of the counties with the most significant number of minks in China, and 60% of the farm families have a herd of minks in some rural villages. Interestingly, June to October is the growth and breeding season for *Haemaphysalis longicornis* tick, the primary vector for SFTSV transmission, in this region. Samples, including intestine, lung, liver, lymph node, brain, spleen, and kidney, were collected from the sick minks in this region and stored at −80°C until use.

### 2.2. Viral Metagenomics (VM)

Multiple displacement amplification (MDA) and metatranscriptomic sequencing (MTT) were used to detect DNA and RNA viruses, respectively [20]. Briefly, we collected the intestine, lung, liver, lymph node, brain, spleen, and kidney from 3 to 5 sick minks, and seven pools were prepared by mixing the same tissues and sent to MDA and DTT for VM separately. All raw reads were quality checked using FastQC version 0.11.7 and trimmed using Trimmomatic version 0.38, and the resultants were used for bioinformatics analyses as clean data. The clean reads of RNA and DNA viromes were respectively mixed and de novo assembled using MEGAHIT version 1.2.9. All contigs were annotated using blastn and diamond blastx searches (*e*-value ≤ 1 × 10^−10^) against refined eukaryotic viral reference database (EVRD) version 1.0 [21]. To verify the authenticity of these virus-like contigs (VLCs), clean reads were mapped back to VLCs, and the vertical and horizontal coverage was determined using samtools version 1.10.

### 2.3. Phylogenetic Analysis

Nucleotide sequences were analyzed using the Neighbor-Joining (NJ) method and Maximum Composite Likelihood model in MEGA11 [22]. Bootstrap values were calculated on 1,000 replicates.

### 2.4. Reverse Transcription Polymerase Chain Reaction (RT-PCR) Assay

RT-PCR were used to detect the viral RNA from mink samples and cell cultures. The procedure was performed as previously described with minor modification [23]. Viral RNA was extracted from the supernatant of mink homogenate and of cultured cells using a TaKaRa MiniBEST Viral RNA/DNA Extraction Kit (TaKaRa, Dalian, China) according to the manufacturer's instructions and then reverse transcribed to cDNA as previously described [24]. DNA was amplified using the MF 5′-TGTTGCTTGTCAGCCTATGAC-3′ and the MR 5′-CAACCAATGATCCTGAGTGGA-3′ with the following conditions: 95°C for 5 min, 35 cycles at 95°C for 30 s, 55°C for 30 s, 72°C for 1 min, and 72°C for 10 min.

### 2.5. Real-Time Quantitative PCR (RT-qPCR)

RT-qPCR were used to measure the Viral RNA levels in mink tissues. Viral RNA extraction and cDNA synthesis were done as described above. RT-qPCR was performed using AugeGreen qPCR Master Mix (US EVERBRIGHT, Tianjin, China) in a 20 *μ*L reaction, including 10 *μ*L qPCR mix, 10 pmol each primer, and 2 *μ*L template, with the primers (qF 5′-GGAGGTGAATCCACCAGAGC-3′ and qR 5′- GTGATTAGCCTCACACCCCC-3′). The following PCR program was used: 95°C for 2 min, 45 cycles of 95°C for 5 s, and 60°C for 30 s. To generate a standard curve, plasmids pMD18-T-SFTSV-pM containing the partial M segment (674 bp) were serially 10-fold diluted from 10^10^ to 10^4^ copies/*μ*L. All RT-qPCR experiments were performed on a LightCycler 96 (Roche) according to the manufacturer's instructions.

### 2.6. Virus Isolation and Observation by Electron Microscopy

Vero and DH82 cell lines, provided by Cell Bank of Chinese Academy of Sciences (Shanghai, China), were used to isolate the viruses. Both the cells were maintained in Dulbecco's modified Eagle's medium (DMEM; GIBCO) supplemented with 10% fetal bovine serum and incubated at 37°C in a 5% CO_2_ tissue culture incubator. The mink tissues (intestine, lung, liver, lymph node, brain, spleen, and kidney) were homogenized in 10% (w/v) sterile phosphate-buffered saline (PBS) and filtered through 0.22 *μ*m membrane filters. The filtrates were added to Vero and DH82 cells and cultured in an incubator for 7–8 days at the conditions as shown above.

For electron microscopy, the supernatant of infected cells was placed directly onto a carbon grid and stained with 2% phosphotungstic acid (PTA; pH 6.8) for 2 min. Then, the grid was examined at 80 kV using an HT7800 transmission electron microscopy (TEM) (Hitachi, Tokyo, Japan).

### 2.7. Indirect Immunofluorescence Assay (IFA)

At 7 days postinfection, DH82 cells were collected and spun onto slides at 1,800 rpm for 5 min using a cell smear centrifuge [25, 26]. Briefly, the slides were fixed and permeabilized with 4% paraformaldehyde (PFA) and 0.5% Triton X-100, respectively, and then were blocked with 3% BSA-PBS and probed with homemade anti-NP of SFTSV antibody and the horseradish peroxidase (HRP)-conjugated antimouse IgG sequentially. DAPI (4′, 6-diamidino-2-phenylindole) was used to stain the nucleus. Images were taken using a Zeiss Axio Observer Z1 microscope (Carl Zeiss Microscopopy GmbH, Oberkochen, Germany).

### 2.8. Western Blot

Western blot was performed as described previously [25, 26]. Briefly, the mock-infected and virus-infected cell lysates were separated by sodium dodecyl-sulfate polyacrylamide gel electrophoresis (SDS-PAGE). Proteins were transferred onto a polyvinylidene fluoride (PVDF) membrane (Cat No. IPVH00010, Merck), which was blocked with 5% skim milk and probed with anti-NP of SFTSV antibody (gifted by Dr. Zekun Wang at Henan University) and the horseradish peroxidase (HRP)-conjugated antimouse IgG sequentially (Cat No. SA00001-1, Proteintech Group, Inc). Signals were visualized by ECL Plus hypersensitive luminescence (Beijing Solarbio Science and Technology Co., Ltd.), and images were developed under a FluorChem E System (ProteinSimple, CA, USA).

## 3. Results and Discussion

**Figure 1:**
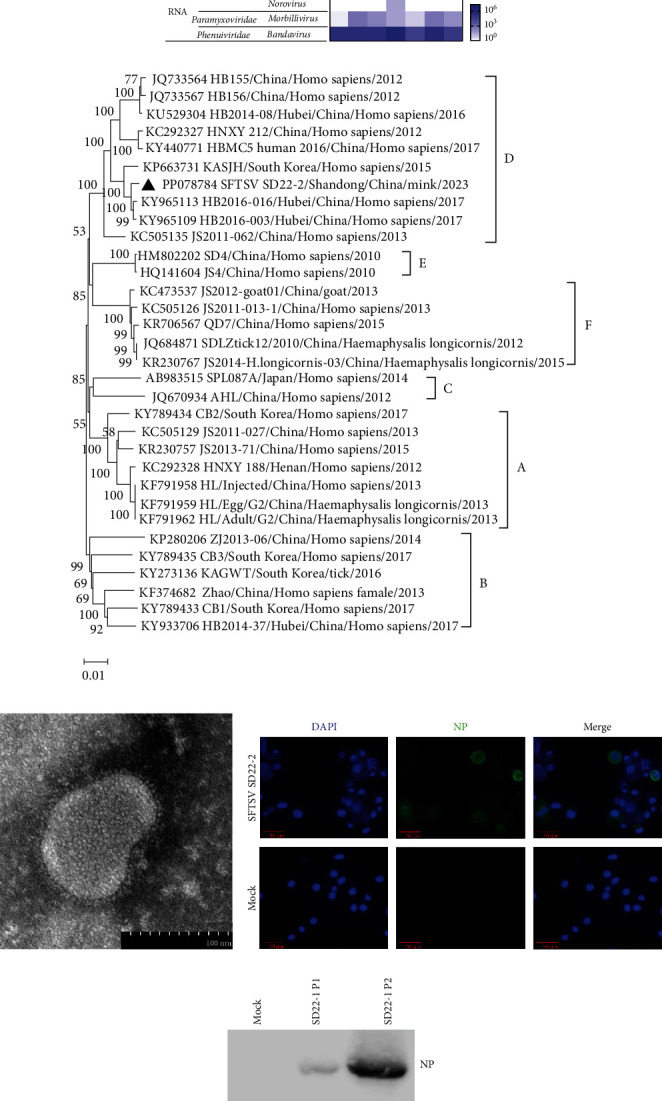
Identification of isolate SFTSV SD22-2 from mink. (a) Heat map showing the different viral reads in seven tissues of minks. Five families of viruses, *Anelloviridae*, *Parvoviridae*, *Caliciviridae*, *Paramyxoviridae*, and *Phenuiviridae*, are found in this study. (b) Phylogenetic analysis and genotype of SFTSV SD22-2 based on the partial L segment sequences (6,237 bp). A solid triangle marks isolated SFTSV SD22-2 in this study. Nucleotide sequences were analyzed using the NJ method and Maximum Composite Likelihood model in MEGA11. Bootstrap values were calculated on 1,000 replicates. (c) Electron microscopy of SFTSV SD22-2. The supernatant of infected Vero cells was placed directly onto a carbon grid and stained with 2% PTA, and the grid was examined at 80 KV using an HT7800 TEM. (d) Detection of the isolate of SD22-2 in DH82 cells by indirect immunofluorescence (IFA). Following 7 days of incubation, DH82 cells were immunostained by IFA using a polyclonal antibody specific for the NP protein of SFTSV and FITC-labeled goat antimouse IgG. (e) Detection of the NP protein by Western blot. At 3 days postinfection, the DH82 cells were lysed for Western blot analysis using anti-NP antibody. Mock was the normal DH82 cells, and SD22-2 P1 and P2 were the infected DH82 cells in passages 1 and 2, respectively.

Bacteria and common viruses were not detected in the sick minks in this study. VM plays a critical role in identifying new viruses. Therefore, MDA and MTT were used to detect unknown DNA and RNA viruses in mink tissues. Combined with the MDA and MTT results, all contigs were assigned to five families, including *Anelloviridae*, *Parvoviridae*, *Caliciviridae*, *Paramyxoviridae*, and *Phenuiviridae*. The viral reads in each pool showed that the lymph node sample had the most abundant viruses, with 2.6 × 10^7^ reads related to the genus *Bandavirus* within the family *Phenuiviridae* (Figure 1(a) and *Supplementary 2*). Previous studies showed that SFTSV is widespread among humans and animals in Shandong, China; and SFTSV antibodies were detected in minks [2, 14, 15]. Therefore, we focus on the SFTSV in the following research. We confirmed the MTT results by RT-PCR for SFTSV described in a previous study [23], and we got the right amplicon of 674 bp from the mink tissues (*Supplementary 1*). Meanwhile, we sequenced the PCR amplicon, and the Sanger sequencing method validated the partial sequence of segment M by MTT (*Supplementary 1*).

To investigate the viral load in different mink tissues, the spleen, liver, and intestine were homogenized in 30% (w/v) sterile PBS and then for RNA extraction and cDNA synthesis. The results of RT-qPCR indicated that there were different viral copies among the three tissues, and the spleen had higher viral loads (8.5 log_10_ RNA/g) than the liver (5.4 log_10_ RNA/g) and intestine (6.6 log_10_ RNA/g; *Supplementary 1*), which was similar to the results of MTT (Figure 1(a) and *Supplementary 2* Table S1). SFTSV infection could lead to the occurrence of viremia and persistent infection in domesticated animals [14], wild animals [27], and humans [28], which may also occur in farmed minks, and the viremia causes SFTSV RNA positive in some organs of minks.

SFTSV has been classified into at least six genotypes according to its genome sequence. We assembled the viral reads and got the partial L, M, and S segments of SFTSV and phylogenetic analyses of the partial L, M, and S segment sequences using MEGA11 with the NJ method (Figure 1(b), and *Supplementary 1*) indicated that the SFTSV obtained from mink in this study belongs to genotype D. In addition, phylogenetic analysis also revealed that the sequences of L and M segments of SFTSV SD22-2 isolate in this study was most genetically close to the HB2016-003 (GenBank accession nos.: KY965109 and KY965092) strain isolated from human serum in 2016 from Hubei, China, with 99.4% and 99.5% similarity and that of S segment of SFTSV SD22-2 isolate was most genetically close to both SDPLP01/2011 (GenBank accession no.: JQ693013) and SDPLDog01/2011 (GenBank accession no.: JQ693003) strains isolated from human and dog in 2011, respectively, with 99.8% similarity. Interestingly, SDPLP01/2011 and SDPLDog01/2011 were isolated from humans and dog, respectively, from Penglai County, which is also in Yantai city [14]. The SFTSV SD22-2S segment showed 99.8% homology with SDPLP01/2011 and SDPLDog01/2011, suggesting a potential link between SFTSV infections among humans and domesticated animals in this region.

For virus isolation, the mink tissues were homogenized and filtered through membrane filters. The filtrates were both added to Vero and DH82 cells for virus isolation, and the cells were cultured at 37°C in a 5% CO_2_ atmosphere with media changes twice a week. The Vero cells infected with the virus did not show apparently cytopathic effect, but virus-infected DH82 cells are differentiated into macrophages with elongated pseudopodia with visible granular particles (*Supplementary 1*). We got an isolate, named SFTSV SD22-2, both from Vero and DH82 cells, and the isolate was confirmed by RT-PCR detection (*Supplementary 1*). Meanwhile, the supernatant of Vero cells exposed to the respective tissue filtrates was placed directly onto a carbon grid, stained with 2% PTA, and examined at 80 kV using an HT7800 TEM. Spherical and enveloped virions, with a diameter of ∼80–100 nm and prominent surface spikes, were clearly observed in supernatant of Vero cells at 60 hr postinfection (Figure 1(c)).

Mock-infected and virus-infected DH82 cells were collected and spun onto slides using a Cytospin centrifuge. After being fixed and permeabilized in 0.5% Triton X-100, the slides were blocked and probed with a primary antibody and a secondary antibody. IFA assay indicated that green fluorescence for NP was detected in infected cells but not in mock-infected cells, suggesting the virus infected DH82 cells (Figure 1(d)). At 5 days postinfection, the virus-infected DH82 cells were lysed for Western blot analysis. As shown in Figure 1(e), NP proteins of SFTSV were detected in the virus-infected DH82 cells but not in the mock cells. Taken together, we successfully isolated and identified an SFTSV strain from minks.

Tick-to-human, human-to-human, and animal-to-human transmissions are the three main routes by which people are infected with SFTSV [13, 29]. There are very few reports on direct contact transmission between animals and animals. This study observed and recorded outbreaks of SFTS-like diseases in minks. When one caged mink suffers the disease and shows clinical symptoms, the minks nearby will show similar clinical symptoms and soon die. Therefore, we hypothesize that mink-to-mink direct contact may transmit the SFTS-like disease. We will infect the isolated SFTSV SD22-2 to minks according to Koch's postulates in the future and determine the SFTSV is the pathogen of the SFTS-like disease in minks.

In this study, we provided evidence that SFTSV was associated with mink death and isolated an SFTSV strain, named SFTSV SD22-2, from farmed mink in Shandong, China. We got the partial sequences of L, M, and S segments of SD22-2, which have been deposited in the GenBank and assigned accession numbers PP078784, PP078783, and PP078785, respectively. Subsequent virus culture, RT-PCR/RT-qPCR, IFA, and Western blot detection of the specific virus in mink specimens confirmed these findings. Our finding showed that minks may be a potential source of SFTSV infection for humans, and we need to conduct a seroprevalence survey on fur-farmed animals that would be a potential risk to public health.

## Data Availability

Data are available at https://www.ncbi.nlm.nih.gov/nucleotide/ with the GenBank accession numbers PP078784, PP078783, and PP078785.

## Supplementary Materials

Supplementary 1 Figure S1: collection site of minks in China. Figure S2: typical clinical signs of minks. Figure S3: RT-PCR results of SFTSV infection in mink samples. Figure S4: MTT results of isolate SFTSV SD22-2 confirmed by Sanger sequencing method. Figure S5: SFTSV RNA quantification in mink tissues by RT-qPCR. Figure S6: phylogenetic analysis and genotype of SFTSV SD22-2 based on the partial M segment sequences (3,290 bp). Figure S7: phylogenetic analysis and genotype of SFTSV SD22-2 based on the partial S segment sequences (1,690 bp). Figure S8: cytopathic effect of the isolate on DH82 cells. Figure S9: RT-PCR results of isolate SFTSV SD22-2 from Vero cells. Figure S10: RT-PCR results of isolate SFTSV SD22-2 from DH82 cells.

Supplementary 2 Different viral reads in seven tissues of minks in this study.
