# Supplementary material for: Isolation and Identification of Severe Fever with Thrombocytopenia Syndrome Virus from Farmed Mink in Shandong, China
Source: Transbound Emerg Dis. 2024 Apr 5;2024:9604673. doi: 10.1155/2024/9604673 (PMC12016913; doi:10.1155/2024/9604673)
Supplement: Supplementary 1 — Figure S1: collection site of minks in China. Figure S2: typical clinical signs of minks. Figure S3: RT-PCR results of SFTSV infection in mink samples. Figure S4: MTT results of isolate SFTSV SD22-2 confirmed by Sanger sequencing method. Figure S5: SFTSV RNA quantification in mink tissues by RT-qPCR. Figure S6: phylogenetic analysis and genotype of SFTSV SD22-2 based on the partial M segment sequences (3,290 bp). Figure S7: phylogenetic analysis and genotype of SFTSV SD22-2 based on the partial S segment sequences (1,690 bp). Figure S8: cytopathic effect of the isolate on DH82 cells. Figure S9: RT-PCR results of isolate SFTSV SD22-2 from Vero cells. Figure S10: RT-PCR results of isolate SFTSV SD22-2 from DH82 cells. [file 9604673.f1.docx]

**Figure S1. Collection site of minks in China.**


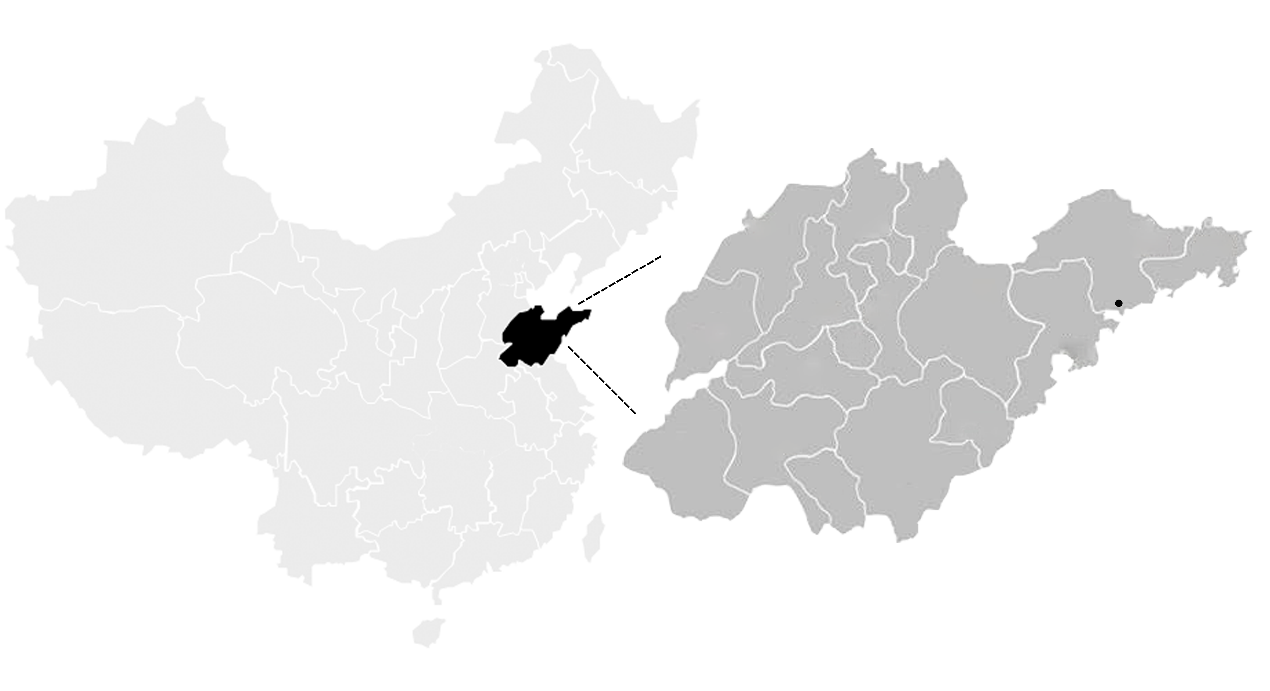
The black dot (●) indicates the site where the mink samples were collected, and it is located in Haiyang City, Shandong Province in eastern China.

**
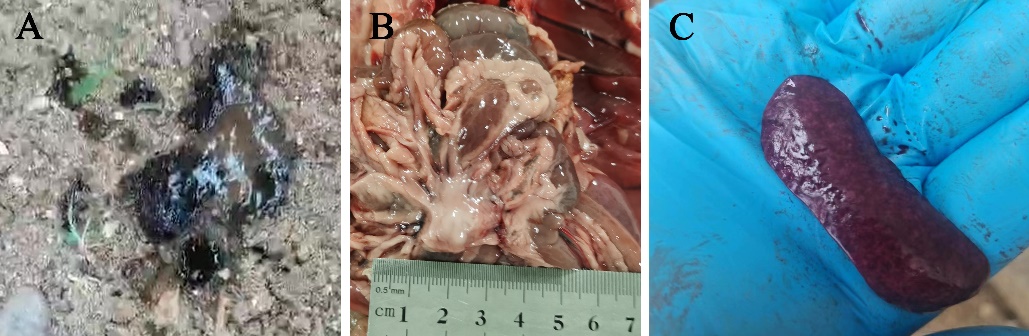
Figure S2. Typical clinical signs of minks.**

(A) Diarrhea with black soft feces. (B) Enlarged lymph nodes and gastrointestinal bleeding. (C) Enlarged spleen with infarction.

**
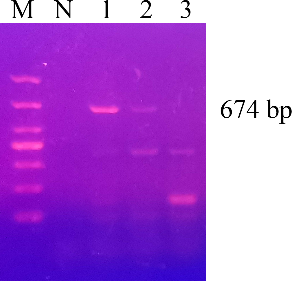
Figure S3. RT-PCR results of SFTSV infection in mink samples.**

RNA of the intestine samples from three minks was extracted, and SFTV was detected through an RT-PCR with the specific primers. Lines 1 and 2 were SFTSV-positive samples, while line 3 was SFTSV-negative. M: DNA Marker DL1000 (TaKaRa). N: Negative control.

**
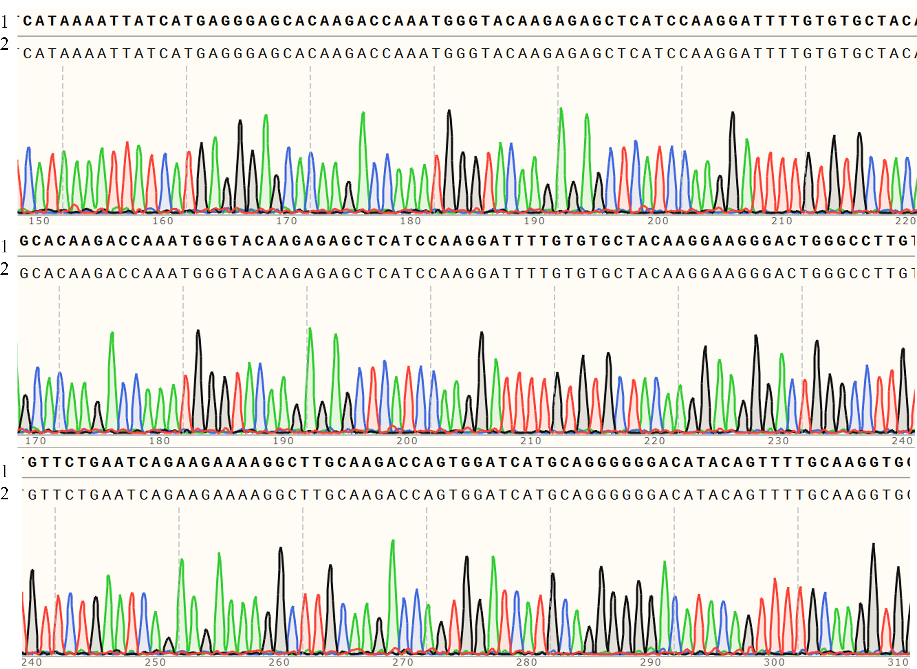
Figure S4. MTT results of isolate SFTSV SD22-2 confirmed by Sanger sequencing method.**

Sequence 1 indicated the results by MTT, and sequence 2 indicated the Sanger sequencing results, which were identical to those of MTT.



**Figure S5. SFTSV RNA quantification in mink tissues by RT-qPCR.**

Viral copy numbers in spleen, liver, and intestine of the sick minks were assessed by RT-qPCR using AugeGreen qPCR Master Mix. Data are presented as mean SEM

**
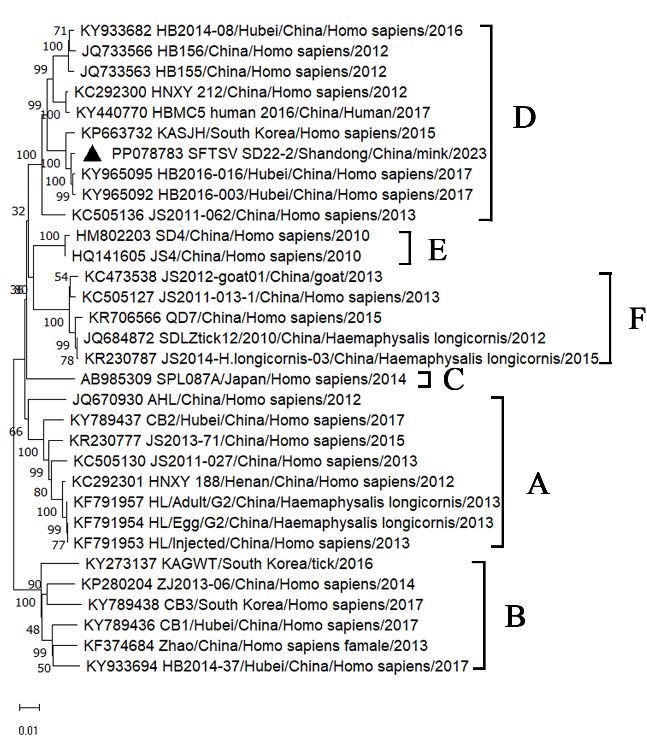
Figure S6. Phylogenetic analysis and genotype of SFTSV SD22-2 based on the partial M segment sequences (3290 bp).**

A solid triangle marks isolated SFTSV SD22-2 in this study. Nucleotide sequences were analyzed using the NJ method and Maximum Composite Likelihood model in MEGA11. Bootstrap values were calculated on 1000 replicates.

**
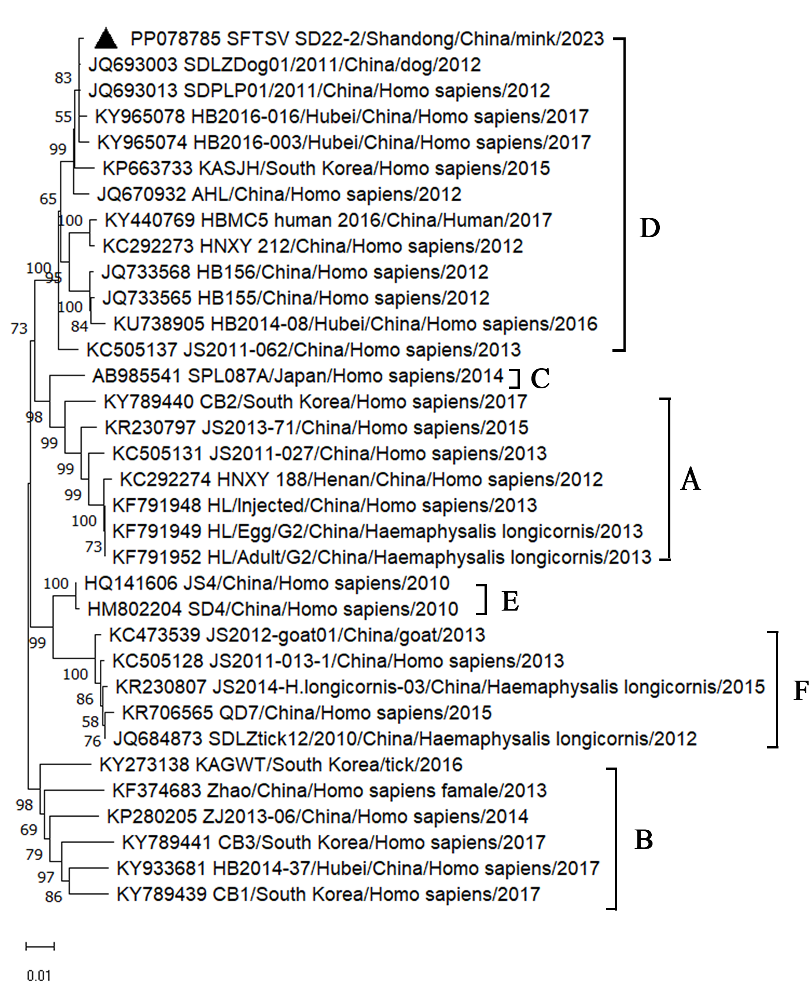
Figure S7. Phylogenetic analysis and genotype of SFTSV SD22-2 based on the partial S segment sequences (1690 bp).**

A solid triangle marks isolated SFTSV SD22-2 in this study. Nucleotide sequences were analyzed using the NJ method and Maximum Composite Likelihood model in MEGA11. Bootstrap values were calculated on 1000 replicates.


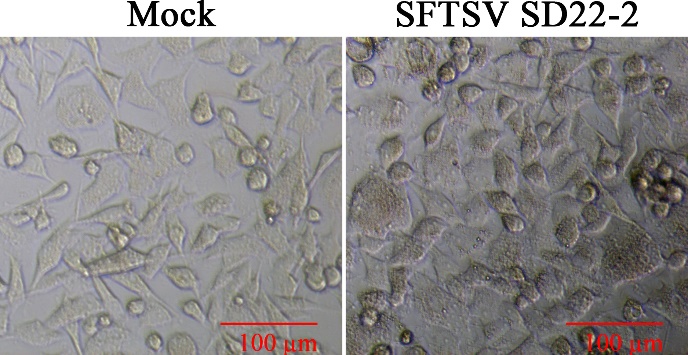
**Figure S8. Cytopathic effect of the isolate on DH82 cells.**

At 4 days postinfection, DH82 cells were shown the typical cytopathic effect of the SFTSV similar to previously described (Yu et al., 2011).

**
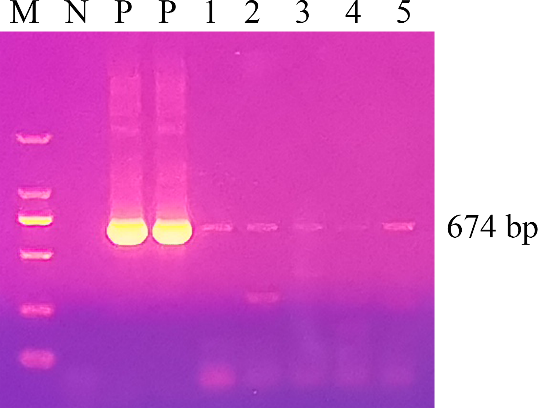
Figure S9. RT-PCR results of isolate SFTSV SD22-2 from Vero cells.**

RNA of the isolate SFTSV SD22-2 from Vero cells was extracted, and SFTV was detected through an RT-PCR with the specific primers. Lines 1, 2, 3, 4, and 5 were SFTSV-positive from passages 1-5 on Vero cells. M: DNA Marker DL2000 (TaKaRa). N: Negative control. P: Positive control (positive plasmid pMD18-T-SFTSV-M).

**
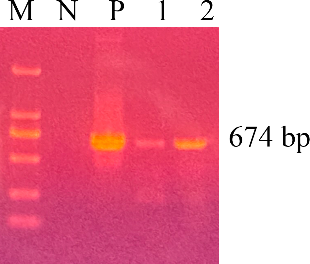
Figure S10. RT-PCR results of isolate SFTSV SD22-2 from DH82 cells.**

RNA of the isolate SFTSV SD22-2 from DH82 cells was extracted, and SFTSV was detected through an RT-PCR with the specific primers. Lines 1 and 2 were SFTSV-positive from passages 1-2 on DH82 cells. M: DNA Marker DL2000 (TaKaRa). N: Negative control. P: Positive control (Positive plasmid pMD18-T-SFTSV-pM).

Table S1 Different viral reads in seven tissues of minks in this study.

| Family | Genus | intestine | lung | liver | lymph node | brain | spleen | kidney |  |
| --- | --- | --- | --- | --- | --- | --- | --- | --- | --- |
| *Anelloviridae* | Unclassified | 13277 | 484505 | 9025973 | 335388 | 3851005 | 13525441 | 18143291 |  |
| *Parvoviridae* | *Amdoparvovirus* | 1681 | 6768 | 222515 | 12183514 | 54939 | 642947 | 25149 |  |
| *Caliciviridae* | *Sapovirus* | 60 | 20 | 0 | 11 | 0 | 0 | 0 |  |
|  | *Norovirus* | 0 | 0 | 0 | 148 | 0 | 0 | 0 |  |
| *Paramyxoviridae* | *Morbillivirus* | 2 | 4219 | 972 | 139 | 12 | 2800 | 416 |  |
| *Phenuiviridae* | *Bandavirus* | 1804003 | 1750822 | 1884042 | 25746121 | 185874 | 3814709 | 299469 |  |

**Reference**

Yu, X. J., Liang, M. F., Zhang, S. Y., Liu, Y., Li, J. D., Sun, Y. L., . . . Li, D. X. (2011). Fever with thrombocytopenia associated with a novel bunyavirus in China. *N Engl J Med, 364*(16), 1523-1532. doi:10.1056/NEJMoa1010095
