# Supplementary material for: Isolation and Identification of Severe Fever with Thrombocytopenia Syndrome Virus from Farmed Mink in Shandong, China
Source: Transbound Emerg Dis. 2024 Apr 5;2024:9604673. doi: 10.1155/2024/9604673 (PMC12016913; doi:10.1155/2024/9604673)
Supplement: Supplementary 2 — Different viral reads in seven tissues of minks in this study. [file 9604673.f2.docx]

Table S1 Different viral reads in seven tissues of minks in this study.

| Family | Genus | intestine | lung | liver | lymph node | brain | spleen | kidney |  |
| --- | --- | --- | --- | --- | --- | --- | --- | --- | --- |
| *Anelloviridae* | Unclassified | 13277 | 484505 | 9025973 | 335388 | 3851005 | 13525441 | 18143291 |  |
| *Parvoviridae* | *Amdoparvovirus* | 1681 | 6768 | 222515 | 12183514 | 54939 | 642947 | 25149 |  |
| *Caliciviridae* | *Sapovirus* | 60 | 20 | 0 | 11 | 0 | 0 | 0 |  |
|  | *Norovirus* | 0 | 0 | 0 | 148 | 0 | 0 | 0 |  |
| *Paramyxoviridae* | *Morbillivirus* | 2 | 4219 | 972 | 139 | 12 | 2800 | 416 |  |
| *Phenuiviridae* | *Bandavirus* | 1804003 | 1750822 | 1884042 | 25746121 | 185874 | 3814709 | 299469 |  |
